# Correlation of CT-based bone mineralization with drilling-force measurements in anatomical specimens is suitable to investigate planning of trans-pedicular spine interventions

**DOI:** 10.1038/s41598-023-50204-2

**Published:** 2024-01-18

**Authors:** Stefanie Wolff, Simon Adler, Elisabeth Eppler, Karin Fischer, Anke Lux, Hermann-Josef Rothkötter, Martin Skalej

**Affiliations:** 1 Clinic for Internal Medicine, Municipal Hospital St. Georg Leipzig, Delitzscher Straße 141, 04129 Leipzig, Germany; 2 Clinic of Neuroradiology, University Hospital Magdeburg, Leipziger Straße 44, 39120 Magdeburg, Germany; 3 Automatisation and Informatics, Harz University of Applied Sciences, Friedrichstraße 57-59, 38855 Wernigerode, Germany; 4 Fraunhofer Institute for Factory Operation and Automation IFF, Sandtorstraße 22, 39106 Magdeburg, Germany; 5 Institute of Anatomy, University of Bern, Baltzerstraße 2, 3012 Bern, Switzerland; 6 Institute of Anatomy and Cell Biology, University of Halle-Wittenberg, Große Steinstraße 52, 06108 Halle (Saale), Germany; 7 Institute of Anatomy, Otto-von-Guericke University Magdeburg, Leipziger Straße 44, 39120 Magdeburg, Germany; 8 Institute of Biometry and Medical Informatics, Otto-von-Guericke University Magdeburg, Leipziger Str. 44, 39120 Magdeburg, Germany; 9 Neuroradiology, Martin-Luther-University Halle-Wittenberg, Ernst-Grube-Straße 40, 06120 Halle (Saale), Germany

**Keywords:** Biophysics, Anatomy, Medical research, Engineering

## Abstract

This interdisciplinary study examined the relationship between bone density and drilling forces required during trans-pedicular access to the vertebra using fresh–frozen thoraco-lumbar vertebrae from two female body donors (A, B). Before and after biomechanical examination, samples underwent high-resolution CT-quantification of total bone density followed by software-based evaluation and processing. CT density measurements (n = 4818) were calculated as gray values (GV), which were highest in T12 for both subjects (GV_maxA_ = 3483.24, GV_maxB_ = 3160.33). Trans-pedicular drilling forces F (Newton N) were highest in L3 (F_maxB_ = 5.67 N) and L4 (F_maxA_ = 5.65 N). In 12 out of 13 specimens, GVs significantly (*p* < 0.001) correlated with force measurements. Among these, Spearman correlations r were poor in two lumbar vertebrae, fair in five specimens, and moderately strong in another five specimens, and highest for T11 (r_A_ = 0.721) and L5 (r_B_ = 0.690). Our results indicate that CT-based analysis of vertebral bone density acquired in anatomical specimens is a promising approach to predict the drilling force appearance as surrogate parameter of its biomechanical properties by e.g., linear regression analysis. The study may be of value as basis for biomechanical investigations to improve planning of the optimal trajectory and to define safety margins for drilling forces during robotic-assisted trans-pedicular interventions on the spine in the future.

## Introduction

Pathological processes of the spine may endanger its functionality as occurs by osteoporotic^1^, degenerative and arthritic processes, e. g., during aging, and by bone destruction after trauma and tumors^2^. Loss of stability and functionality of the bone further strongly affect quality of life because they may cause severe pain and neurologic symptoms like paraplegia by narrowing the spinal canal^3^. Standard therapies of different diseases of the spine are surgical decompression and stabilization. Nevertheless, therapeutic interventions by open surgery may require a long period for post-surgical recovery and relief from symptoms, and can even further impair quality of life. Therefore, and especially for general patient conditions such as e. g., advanced age, reduced general physiology, and overall unfavorable prognosis, minimal-invasive local procedures are increasingly applied to reduce physical effort and immobilization time for the patients. The trans-pedicular, image-based approach to the vertebra for procedures such as insertion of screws^4–6^ and other devices and material during kypho- and vertebroplasty, spondylodesis and radiofrequency ablation^7,8^ as well as for diagnostic bone biopsy is frequently applied^9^. Robotic assistance is increasingly evolving because of general advantages like precision, mechanical power and speed^10,11^.

However, strong forces are required to penetrate the calcified bone, and the physical properties of different osseous tissues are complex. Automatic and even semi-automatic robotic-assisted trans-pedicular treatment requires the validation between the planned intervention and the actual conditions. Robotic systems are equipped with capabilities to measure actual forces at their end-effector. For validation, it is required to derive expected forces from pre-interventional imaging. During robotic-assisted intervention, currently measured and expected forces can be compared and used to support the surgeon.

This interdisciplinary study aimed at creating a basic model for biomechanical investigations to improve planning of the optimal trajectory through the vertebral arc during robotic-assisted trans-pedicular interventions on the spine in the future. For that purpose, the forces during bone drilling were recorded on human cadaver samples, and data were compared with bone density parameters achievable from pre-interventional CT-measurements.

## Materials and methods

### Study samples

The samples comprised two female body donors, pre-designated in the frame of the institutional body donation program for the Institute of Anatomy of the Otto-von-Guericke University Magdeburg with relevant pre-approvals for educational and research studies. Both body donors of the present study have individually given written informed consent, which explicitly included the post-mortem use of the body for experimental morphology research. The requirement for ethics approval was deemed unnecessary according to relevant regulations for this research project by the institutional ethics committee (Ethik-Kommission der Otto-von-Guericke-Universität an der Medizinischen Fakultät und am Universitätsklinikum Magdeburg A.ö.R.). Due to data protection regulations in Germany, the Institute of Anatomy has no access to the patient data of the body donors. The death certificate provided no information about diseases of the skeleton. Body donor A, aged 73 years, had a normal body mass index (BMI) of 22.23, and donor B, aged 87 years, had a low BMI of 14.69 (Table 1).

**Table 1:** Characteristics of the body donors and samples.

Individual	Sex	Age at death	Height [m]	Weight [kg]	BMI [kg/m^2^]	Freezing time [months]
A	f	73	1.59	56.2	22.23	16
B	f	87	1.65	40.0	14.69	6.5

### Sample processing

**Figure 1:**
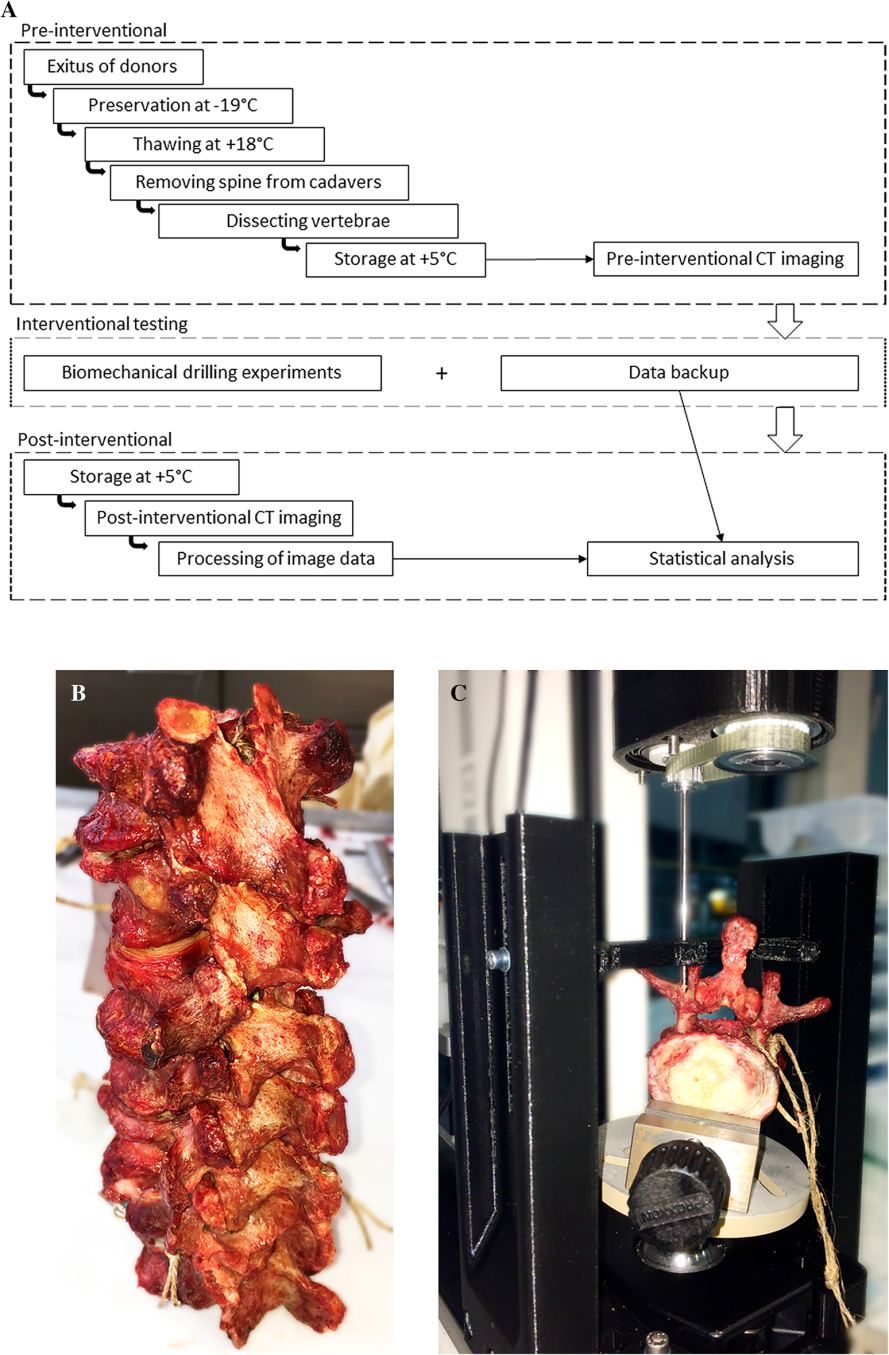
(**A**) Flowchart of overall procedure*.* (**B**) Dissected spine from thoracic vertebra T11 to L5 from individual B, left dorsal view. (**C**) Lumbar vertebra during trans-pedicular drilling using a motorized material testing machine (Mark-10 Force Test Stand ESM301) as previously described^14^, supplied for the present study with clinical drilling tools (ARROW OnControl Bone Lesion Biopsy System Tray).

Sample processing was carried out strictly in accordance with the relevant standard guidelines and regulations of the anatomy department (Fig. 1A). In brief, bodies were preserved at − 19 °C, i.e., the optimal temperature for bone storage to prevent any putrefaction/decomposition, so that after thawing, they can be safely used for mechanical testing at least for storage periods of up to one year as previously described^12^. There was no manipulation or chemical impact due to any other handling or substances. After the respective freezing times of 6.5 and 16 months (Table 1), the cadavers thawed by placement at + 18 °C for six days. Thereafter, the spinal columns were excised from the levels above thoracic vertebra T11 to below lumbar vertebra L5 by an experienced anatomist. Henceforth, human vertebrae samples (n = 14) were stored at + 5 °C between the different preparation steps (Fig. 1A), including removal of soft tissue to avoid entangling with the drilling device. Special emphasis was laid on precise dissection of the pedicle entrances (Fig. 1B,C), as well as on gentle handling to avoid any biomechanical stress and preserve the mechanical integrity of the vertebral neural arch. Each specimen was carefully inspected for intactness from prior fractures and implants as well as post mortem lesions, which would lead to exclusion of the specimens. One specimen (T11 from individual B) was excluded due to complete disruption in several fragments during physical penetration.

### Biomechanical testing

After defrosting for in total twelve days, three hours prior to biomechanical testing, the samples were removed from the refrigerator and acclimated to room temperature at + 18 °C (± 0.5 °C) as test condition (Fig. 1A). All experiments were performed using a motorized material testing machine (Mark-10 Force Test Stand ESM301, Mark-10 Corporation, NY), programmed to move a force gauge (G) (Mark-10 M5-20 Force Gauge*)* at a constant velocity of 2 mm/s corresponding to 120 mm/min, which was within the standard speed range of 13–330 mm/min^13^, as previously established^14^. For the present study, the motorized test device was combined with clinical drilling tools (ARROW OnControl Bone Lesion Biopsy System Tray, 11G 15 cm Access/13G 19 cm Biopsy, Teleflex Medical, Ireland) installed to the force gauge. To ensure comparable test conditions, for each vertebra, new drilling tools were used. We examined the displacement-controlled axial force in Newton (N) to maintain a constant penetration speed of 2 mm/s at a set constant drilling speed of 430 R/min and a nominal torque of 2.25 Nm (Table 2). These defined settings were applied to ensure the reproducibility of the experiments in the future and refer to previous investigation^14^. In brief, specimens were attached on a horizontal steel platform based on a ball joint, which allowed tilting/small “settling” movements of the vertebrae in all directions (Fig. 1C). At the beginning of each measurement, the drilling tool moved downwards to enter the vertebra until the drilling measurement was terminated manually. The aim was to access the vertebral body by drilling through the cortical shell of the neural arch and the internal part of the pedicle. The pedicle’s point of entry had been previously determined based on the records of Roy-Camille and co-workers^15^. The process forces during the interventional testing were recorded for further analysis.

**Table 2:** Technical parameters for drilling experiments.

Voltage	5 V
Velocity	2 mm/s
Nominal speed	430 R/min
Nominal torque	2.25 Nm
Measurement frequency	1 kHz

### Imaging

For analysis of the drilling hole voxels as described below, mineral bone densities of the vertebra specimens were measured prior to and after the interventional tests by high-resolution spiral computed tomography (CT) with a SOMATOM Definition AS + scanner (Siemens Healthcare GmbH, Erlangen, Germany) using standard settings of the manufacturer (Table 3). The CT-based measurements resulted in Hounsfield units, which have been demonstrated to be suitable to predict regional bone mineral density in lumbar CT^16^. For that purpose, each vertebra (T11—L5) belonging to an individual were aligned in anatomical order (Fig. 1B), and cranio-caudally CT-scanned as transverse sections with a slice thickness of 0.6 mm. Final reconstruction was performed with a high-resolution kernel (B60s) using specific technical parameters (Table 3). The acquired images were stored as DICOM (Digital Imaging and Communications in Medicine)^17^ files for further use.

**Table 3:** Technical parameters of the CT scanner.

Voltage	80 kV
Milliampère-second product	55/959 mAs
CT Dose index	1.02–1.04 mGy
Dose-length product	45–51 mGycm
Integration time	1.0 s
Slice thickness	0.6 mm
Voxel size	0.3–0.6 mm
Resolution of the CT images	512 × 512

### Software-based processing of the image data

The CT-based image data were processed using MeVisLab, version 3.1.1 (MeVis Medical Solutions AG, Bremen, Germany). The 1st step, “*Register”* was the (image) registration, also termed *“fusion”*, bringing the pre- and post-interventional CT image data into spatial concordance (positioning and orientation), performed by a manual pre-processing step and an automated post-processing registration. During the 2nd step, termed *“set drilling pathway”,* the trans-pedicular drilling pathway was segmented to separate the region of interest from less relevant areas. In a first approach, we estimated the drilling pathway from the difference between the pre- and post-interventional images using a principal component analysis as previously described^14^.

**Figure 2:**
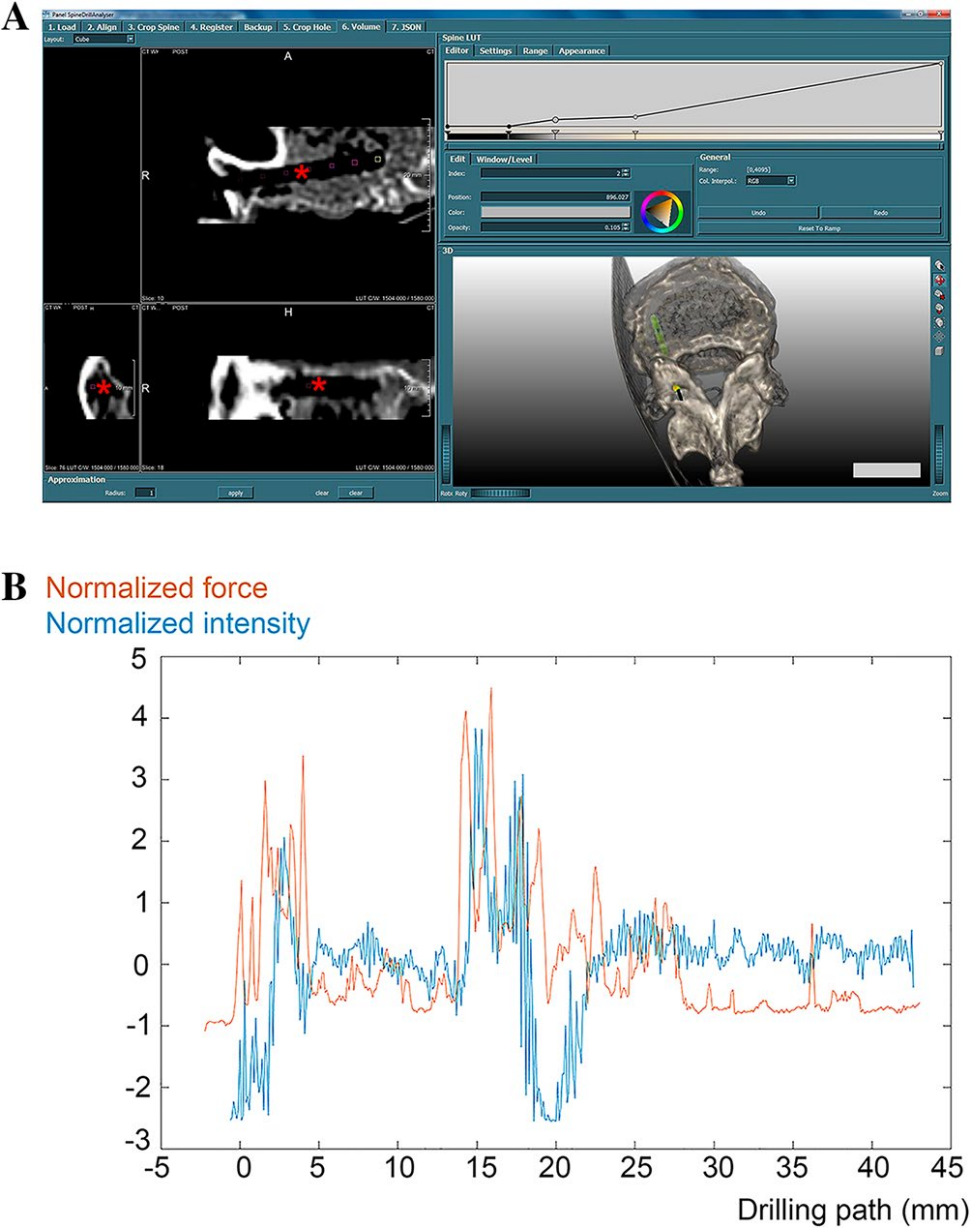
(**A**) Program interface of MevisLab (https://www.mevislab.de) with data of vertebra T12 from individual B (own illustration). Left image: DICOM slice view of set drilling pathway of 11G drill in three orthogonal slice views. Right image: 3D volume rendering (bottom) using LUT (lookup table) function (top). Red asterisk depicts the drilling canal with a length of 29.9 mm (compare Table 4) and a diameter 3.05 mm. 3D-bar: 25 mm. (**B**) Normalized data sets of intensity and force values (y-axis) in vertebra L3 from individual A. Blue line: normalized intensity values. Red line: normalized force values. For better overview, intensity and force intervention paths after interpolation as described above along the drilling path (x-axis, mm).

The estimation of the start- and end-points depends on the image resolution and the artifacts during image segmentation. As previously described, definition of start- and end-points at sub-voxel precision is difficult due to artifacts during segmentation and the small size of the drilling pathway^14^. With an additionally created tool we enabled users to manually fine-tune the start- and end-positions to achieve sub-voxel precision of the drilling pathway in all orthogonal (sagittal, coronal and axial planes) slice views and in an interactive 3D view (Fig. 2A). The 3rd experimental step, termed “*Show drilling pathway”,* based on the knowledge of the outer diameter of the hollow drilling tool, i.e. 11G corresponding to 3.05 mm. From that, a virtual cylindrical segmentation mask was generated as equivalent to the drilling pathway as previously described^14^. This mask demonstrated, based on the pre-interventional scan, the bone densities along the drilling pathway in color-encoded form (Fig. 2A). The next step, designated as “*Modify”,* was the translation of the Hounsfield Units (HU) into gray values (GV) by the formula:

$$\begin{array}{*{20}c} { - 1^{\prime } 025{\text{ HU}}\, = \,1{\text{ GV}}} \\ {1{\text{ HU}}\, = \,1/\left( { - 1^{\prime } 025} \right){\text{ GV}}} \\ \end{array}$$

Finally, by the step “*Transform”* the pathway’s densities were extracted by software-based transforming. Transformation along the x-axis, termed *Shift*, was required in some cases. Since the two-dimensional force-drilling pathway curve (approximately 0–4 Nm) and the resulting four-dimensional (x-, y-, z-coordinates and intensity value) CT-based intensity-pathway curve of the same vertebra (GV 0–4´096) were located within different value ranges, both data sets had to be normalized. For that purpose, both data sets were aligned to the point of insertion where intensities for the first time changed, and the driller had first contact to the bone yielding a first increase in linear forces (Fig. 2B). Image processing was performed using Adobe Photoshop 21.2.0 (Adobe Inc., San José, CA).

### Statistical analysis

Association between the measured parameters was assessed by the Spearman’s rank correlation coefficient (nonparametric). Correlation coefficients (*r*) were computed for all specimens, and separated by region (thoracic T and lumbar L), by individuals A and B, by both, regions and individuals, and in total. Adapted for the present study from Chan^18^, correlation values r < 0.3 were classified as poor, between 0.3 and < 0.6 as fair, between 0.6 and < 0.8 as moderately strong and ≥ 0.8 as very strong. Furthermore, scatterplot diagrams were created based on Pearson correlation (parametric), and the square of Pearson correlation coefficient R^2^ (coefficient of determination) was calculated for agreement of measured variables with those predicted from linear regression analysis, i.e., the proportion of variance in either variable which is accounted for by the other^19^. For all calculations, *p*-values (significance level) of 0.01 or less were considered as statistically significant. Arithmetical correlation analyzes were generated using Microsoft Excel 2019 MSO (Version 1909, Microsoft Corporation, Redmond, WA) and IBM SPSS Statistics 26 (IBM Corporation, Armonk, NY).

## Results

### Technical success

One out of the 14 specimens (T11 from individual B) was excluded from further examinations due to its complete disruption during physical penetration. According to the post-interventional CT image data, the drilling tool placements were successful in all other cases since there were no perforations of the spinal canal nor any other unexpected mal-positioning. Length of the drilled pathway ranged between 29.3 and 46.0 mm and expectedly differed between thoracic (29.3–36.2 mm) and lumbar (30.9–46.0 mm) vertebrae (Table 4).

**Table 4:** Spearman’s rank correlation coefficient (nonparametric) separated by vertebrae. All correlation coefficients (r < 0.3 classified as poor, between 0.3 and < 0.6 as fair marked with *, between 0.6 and < 0.8 as moderately strong marked with **) were significant at *p* < 0.001, except for L1 from individual A. Square of Pearson correlation coefficient R^2^ (coefficient of determination) separated by vertebrae (for individual scatterplots see Supplementary material 1 and 2). T = thoracic vertebra, L = lumbar vertebra, n = number of pairs used for calculation along the drilling pathway (length in millimeters, mm).

	Correlation coefficient *r*	Coefficient of determination R^2^	Significance level *p*	n	Length of drilling pathway [mm]
Individual A					
T 11	0.721**	0.521	< 0.001	295	36.2
T 12	0.687**	0.291	< 0.001	300	29.3
L 1	0.107	0.020	.051	330	32.6
L 2	0.524*	0.227	< 0.001	429	46.0
L 3	0.512*	0.350	< 0.001	436	43.0
L 4	0.469*	0.281	< 0.001	414	40.1
L 5	0.542*	0.343	< 0.001	416	40.5
Individual B					
T 12	0.686**	0.290	< 0.001	309	29.9
L 1	0.400*	0.140	< 0.001	418	41.7
L 2	0.204	0.029	< 0.001	287	30.9
L 3	0.202	0.042	< 0.001	398	39.1
L 4	0.659**	0.520	< 0.001	385	38.3
L 5	0.690**	0.137	< 0.001	401	39.1

### Biomechanical testing of drilling forces

**Figure 3:**
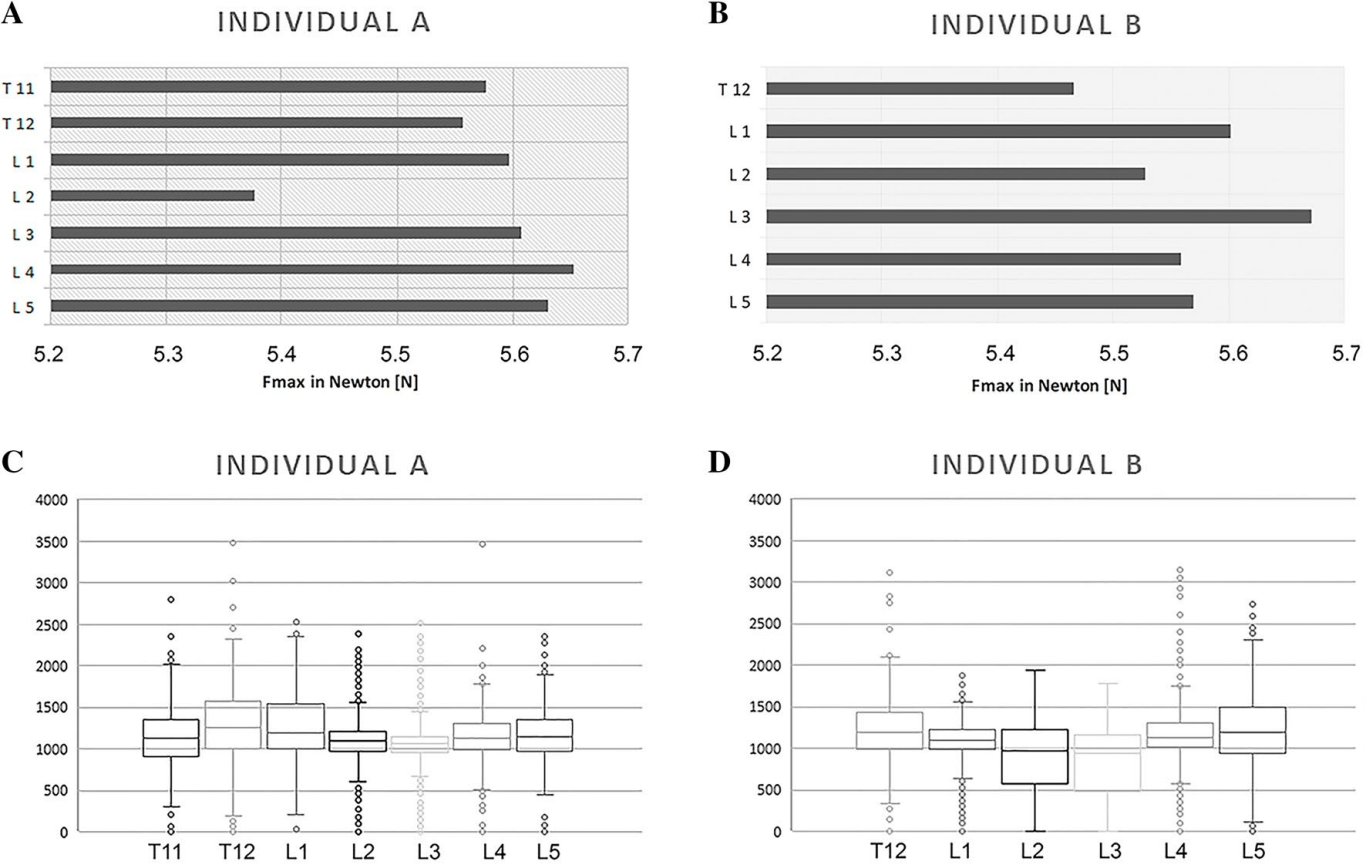
(**A, B**) Maxima of force measurement data (F_Max_) along the drilling pathways (x-axis, N) of vertebrae (y-axis) in (**A**) individual A and (**B**) individual B. (**C, D**) Totality of GV of the drilling pathway (y-axis) in the examined vertebrae (x-axis) of (**C**) individual A and (**D**) individual B using box-plots. A single box is limited by the 25%- and 75%-quartile. Whisker depicts the remaining quartile and reflects maxima and minima. Line inside the box indicates the median value.

Based on the described technical settings (Table 2), the following force data were obtained: The measured forces were highest (Fig. 3A,B) for individual A in the drilling pathway of vertebra L4 (F_maxA_ = 5.65 N, Fig. 3A), and for individual B in the drilling pathway of vertebra L3 (F_maxB_ = 5.67 N, Fig. 3B). The mean values of the total force measurements were F_meanA_ = 4.754 N in individual A, and F_meanB_ = 4.191 N in individual B (data not shown).

### Processing of CT image data

By software-supported subtraction of the post-interventional CT-data from the pre-interventional image data as described above, it was possible to map only the GVs of the drilling pathways, which were analyzed using boxplots (Fig. 3C,D). Maximum, minimum and arithmetic mean of the GVs were determined from all recorded intensity values within a range of ± 0.2 mm around a single intensity value. Using this so-called low pass filter, a range of 0.4 mm was recorded.

The maximum GV was found in the drilled pathway of vertebra T12, both in subject A (GV_maxA_ = 3483.24, Fig. 3C) and in subject B (GV_maxB_ = 3160.33, Fig. 3D). Based on these new data sets, stronger correlation coefficients of the intensity value maxima with the identical force values were obtained as follows.

### Correlation between drilling forces and CT imaging data

In total n = 4818 sample measurements were achieved along the different drilling canals (Table 4). GVs significantly (*p* < 0.001) correlated with force measurements in 12 out of the 13 specimens inspected (Table 4), except for L1 of individual A (r = 0.107). For all thoraco-lumbar vertebra specimens together, the Spearman correlation was moderately strong (r = 0.466). In detail, Spearman correlations were poor in two lumbar vertebrae, fair in five specimens, and moderately strong in another five specimens, respectively (Table 4), with the highest r for T11 in individual A (r = 0.721) and L5 in individual B (r = 0.690). Square of Pearson correlation coefficient R^2^ (coefficient of determination) ranged between 0.140 (L1 in individual B) and 0.521 (T11 in individual A, Table 4).

### Correlations between drilling forces and CT imaging data analyzed by individual and vertebra region

**Figure 4:**
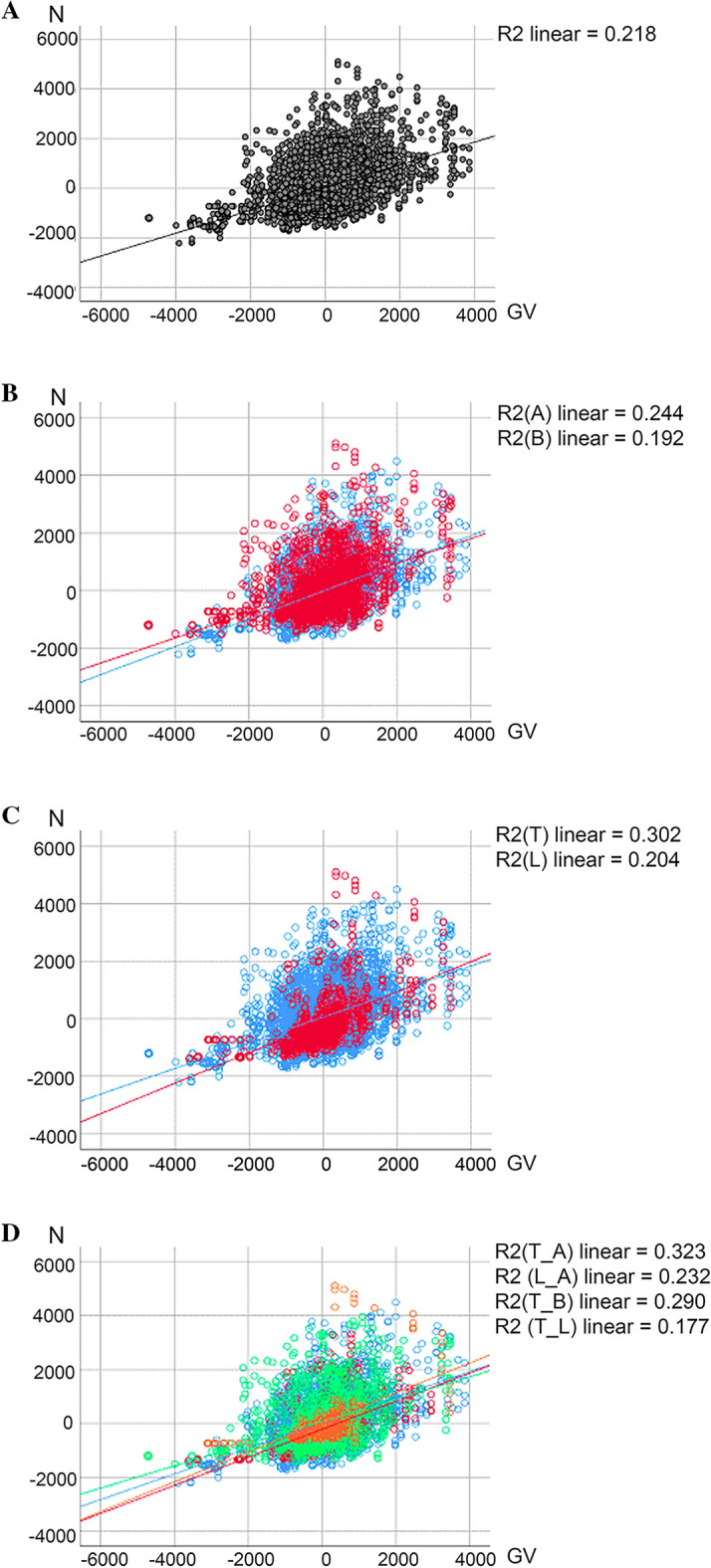
Scatterplot diagrams of Pearson rank correlations (parametric) of normalized force values (N, y-axis) and intensity values (GV, x-axis). (**A**) All values analyzed together. (**B**) Correlations sorted by individual (A: blue, B: red). (**C**) Correlations sorted by vertebra region (thoracic T: red, lumbar L: blue). (**D**) Correlations sorted by both, individuals and regions combined (T_A: red, L_A: blue, T_B: orange, L_B: green).

Spearman correlations between drilling forces and intensities from CT imaging data were further analyzed by individual, by vertebra region, as well as for both combined (Table 5). Correlation coefficients r were fair for individuals, i. e., individual A (r = 0.472) and individual B (r = 0.457, Table 5). Correlations between mechanical strength of the neural arch and CT imaging data tended to be higher in the thoracic (r = 0.665), than in the lumbar vertebrae (r = 0.434, Table 5), however, one has to consider the different number of data pairs achieved along the drilling canal, which were lower in the thoracic (n = 904) than in the lumbar region (n = 3914). When calculating for both, the vertebra region and the individual combined, correlation coefficients were highest for A_T (r = 0.687) and B_T (r = 0.686), and lowest for A_L (r = 0.430). All correlation coefficients were highly significant (*p* < 0.001, Table 5). Pearson linear regression analyzed for each vertebra separately (Supplementary material 1 and 2) revealed a coefficient of determination of in total R^2^ = 0.218 (Fig. 4A), for individual A in all vertebrae R^2^ = 0.244, for individual B in all vertebrae R^2^ = 0.192 (Fig. 4B), for the thoracic region R^2^ = 0.302, for the lumbar region R^2^ = 0.204 (Fig. 4C), and sorted by region (T, L) and individuum (A, B) for T_A R^2^ = 0.323, for L_A R^2^ = 0.232, for T_B R^2^ = 0.290, and for T_L R^2^ = 0.177 (Fig. 4D).

**Table 5:** Spearman’s rank correlation coefficient (nonparametric) according to anatomical regions, individuals, and both together. Correlation coefficients (r < 0.3 classified as poor, 0.3 ≤ r < 0.6 as fair, marked with*, 0.6 ≤ r < 0.8 as moderately strong, marked with **) computed according to thoracic (T) and lumbar (L) region, according to individuals A and B, as well as according to both, individuals and regions (A_T, A_L, B_T, B_L). All correlation coefficients were significant at *p* < 0.001. n = number of pairs used for calculation along the drilling pathway.

	Correlation coefficient *r*	Significance level *p*	n
Region			
T	0.665**	< 0.001	904
L	0.434*	< 0.001	3914
Individual			
A	0.472*	< 0.001	2620
B	0.457*	< 0.001	2198
Individual_Region			
A_T	0.687**	< 0.001	595
A_L	0.430*	< 0.001	2025
B_T	0.686**	< 0.001	309
B_L	0.437*	< 0.001	1889

## Discussion

### Study samples and processing

In the present study, we investigated in lower thoracic and the lumbar vertebrae, which are typical sites of bone metastases^2,3^, the axial penetration forces required for access to the arch and body in trans-pedicular needle drilling. Regarding the two aged female body donors, the investigation of age or sex dependence of the consistency of osseous tissues was beyond the scope of the present study as we have outlined in the limitation section. Due to the data protection regulations, we were not able to correlate the present results to putative diseases. The bodies were preserved for 6.5 and 16 months, respectively, at − 19 °C, which is presumed to be the optimal temperature for bone storage to prevent any putrefaction/decomposition. Few studies have compared frozen bone tissue and bones after fixation in 4% buffered formaldehyde. In terms of drilling, no marked differences have been reported in fresh, frozen or fixed bone tissue as previously described^20^. For instance, bones after thawing were assumed to be safely used for mechanical testing at least for storage periods of up to one year, which was the maximum time investigated in a long-time study^12^, and the use of fresh–frozen bone specimens is recommended in biomechanical studies investigating failure loads of orthopedic implants^21^.

### Technical success and limitations

The present experimental setting aimed at mimicking the interventional setting by cleaning the bone from soft tissue, which might entangle with the drilling device. The drilling procedure was performed similar to previous report^14^, which used a drilling velocity of 2 mm/s. Most recently, a biomechanical study used a similar setting for drilling of the spine in minipigs^11^. Although drilling-processes have already been investigated^22^, the presented correlation with modern CT-based imaging in human bone is promising as previously outlined^14^, e. g., to contribute to improved planning of the optimal trajectory to increase screw purchase^23^.

A limitation of the study is that the vertebrae may not be representative for diseased osseous tissue, although we deliberately did not exclude vertebrae with signs of pathological disease, which may explain complete disruption of one vertebra during physical penetration. Similarly, higher perforation rates were observed in a cadaveric study where all specimens had degenerative changes in the vertebrae and osteoporosis^6^. Therefore, data interpretation should be with caution since it may be difficult to extrapolate that the sensitivity achieved with our processing will be sufficient to detect a transition in the drilling canal from healthy to pathological osseous tissue. Further, as outlined above, the present study was limited to some samples from only two individuals, however with advanced age as is typical for many patients undergoing minimal invasive therapies. Despite these limitations, some information may be drawn from these experiments. First of all, the fresh–frozen vertebrae, which according to previous studies^12,24–30^ should not be altered in their biomechanical properties, were suitable for the drilling experiments and correct placement of the devices as well as for CT-based imaging. Bone drill quality should be further explored^31^ and anatomical osseous specimens as demonstrated in the present study may potentially be suitable for investigation.

### Correlation between process forces and CT imaging data

Correlation analyses of the normalized data sets revealed that increase in force was accompanied by elevated intensities in both individuals and almost all vertebrae. Sample size and selected vertebrae of the present study were not sufficient to relate the cases to one or multiple reasons. Further research is required to collect and cluster cases to identify explanatory characteristics. The above-mentioned increase in force in our study applied in particular to the entry point, which corresponds to the passage through the cortical bone. This may suggest that the cortical bone significantly contributes to maintain the mechanical integrity of the vertebral arch and body. This is in agreement with the specific histological arrangement of the bone lamellae^26^. Furthermore, in human tibiae, cortical bone thickness and trabecular structure density were proposed to account for 90% of the mechanical holding stability of internal fixator screws^32^.

Technical support systems could monitor whether change in drilling force would be expected on the respective path and safety measures could be activated in case of an unpredicted event, for example by altered bone density. Most recently, a prototype high-speed drill with a haptic interface was able to accurately detect the penetration of the posterior lamina in spine surgery in miniature pigs^11^. Prediction of absolute forces from HU depends on many factors. Compared to classical interventions, which penetrate the bone by hammering or drilling, respectively, robotic-assisted surgery reduces translational forces by drilling. Drilling forces depend not only on rotational speed, but also on geometry of the driller (e.g., number of rims, angles of edges), its sharpness and, also on the properties of the already removed tissue which temporarily remains within the drilling hole.

It is also known from bone mineral density measurements that there is a high variability between individuals and different anatomical sites, and the cancellous bone possesses an irregular trabecular structure^1^. In another approach to improve preoperative planning and intraoperative navigation systems, a novel drilled surface imaging technique was applied by comparing anatomical integration property and contact bone volume of the screws implanted along the trans-pedicular trajectory and the cortical bone trajectory, and their surrounding tissues^33^. Better knowledge of general geometrical and individual features, which may be of clinical relevance, is necessary^34^. Furthermore, surfaces of the vertebrae are not perpendicular to the drill and uneven so that an offset must determine the exact moment when force measurements must be aligned with image data. While posterior element morphology has been proposed to be highly variable and largely unpredictable from other vertebral dimensions^35^ and experimental investigation of stability and fracture may not be completely transferable, biomechanical ex vivo experiments may at least demonstrate tendencies^36^. In that recent study in miniature pigs, the reaction time to detect penetration and the distance travelled after penetration were significantly improved by a surgical drill with a haptic interface when compared with a handheld surgical drill without the haptic interface penetration detection function^11^.

Quantitative CT has been demonstrated to predict the tension of failure of functional segments of lumbar vertebrae^37^. The present correlations indicate that predictions may be possible, but more detailed analyses are required such as selecting the average for a path segment. When analyzed as adapted from Chan^18^ and Cohen^19^, only L1 in individual A, and L2 and L3 in individual B showed no correlation despite the higher number of data pairs for lumbar vertebrae.

In a recent study in cadaveric lumbar vertebra specimens, cementation of fenestrated pedicle screws increased overall pullout forces, which were correlated with plume diameter, vertebral body width, and angle of screw insertion, however with an unclear relationship between the geometric properties of the cement plume and the overall strength of the screw-bone interface. This may point to a role of the width of the vertebral body not alone in the pull-out forces in that study^38^, but maybe also in the drilling forces of the present study, however more studies are required.

Nevertheless, the drilling paths were selected by the investigators and manually performed. So far, data are conflicting with regard to free-hand screw placement versus CT-guided procedures as reviewed by Chan and co-workers^39^. Accuracy of intraoperative CT-guided integrated instrumentation have been demonstrated to exceed other guidance systems, even 3D-C-arm stereotactic navigation^40^. CT-guided interventions have been demonstrated to reduce risks of mal-positioning and should be further explored using larger scale studies^41^. The present study supports this assumption and may encourage more anatomical specimen-based CT-investigations to complement patient treatment-acquired data by a broader scientific basis of the spine^42^ and extrapolation of data^34^.

### Limitations

This biomechanical in vitro study inherits some limitations, in particular the limited number of specimens (in total 13) from two aged female body donors. Looking for potential sex differences or comparison with samples from younger donors was beyond the scope of the present study. Further, as outlined above, the vertebrae obtained from aged body donors may not be representative for diseased bone tissue. Further, the study was limited to thoracic and lumbar vertebrae, which should be expanded to all levels of the spine. Correlations were fair or moderately strong, nevertheless, our findings may be helpful for planning future in vitro studies.

## Conclusions

The results of this study indicate that CT-based analysis of vertebral bone density acquired in anatomical specimens is a promising approach to further study the drilling force appearance as surrogate parameter of its biomechanical properties. We are aware that up to now, there is a quite far distance from any clinical applications, but our study may be of value as basis for biomechanical investigations to improve planning of the optimal trajectory and to define safety margins for drilling forces during robotic-assisted trans-pedicular interventions on the spine in the future.

### Supplementary Information

Supplementary Information 1.

Supplementary Information 2.

## Data Availability

The datasets generated and analyzed during the current study are available from the corresponding author upon reasonable request.

## References

[1] Lochmüller E-M (2002). Mechanical strength of the thoracolumbar spine in the elderly: Prediction from in situ dual-energy X-ray absorptiometry, quantitative computed tomography (QCT), upper and lower limb peripheral QCT, and quantitative ultrasound. Bone.

[2] Pagnotti GM, Thompson WR, Guise TA, Rubin CT (2021). Suppression of cancer-associated bone loss through dynamic mechanical loading. Bone.

[3] Ryan C (2022). Epidemiology of bone metastases. Bone.

[4] Konermann W, Haaker R (2003). Navigation und Robotic in der Gelenk- und Wirbelsäulenchirurgie.

[5] Peh S (2020). Accuracy of augmented reality surgical navigation for minimally invasive pedicle screw insertion in the thoracic and lumbar spine with a new tracking device. Spine J. Off. J. N. Am. Spine Soc..

[6] Sallent A (2019). Precision and safety of multilevel cervical transpedicular screw fixation with 3D patient-specific guides; A cadaveric study. Sci. Rep..

[7] Gazis AN, Beuing O, Franke J, Jöllenbeck B, Skalej M (2014). Bipolar radiofrequency ablation of spinal tumors: Predictability, safety and outcome. Spine J. Off. J. N. Am. Spine Soc..

[8] Gazis A, Beuing O, Jöllenbeck B, Franke J, Skalej M (2012). Bipolar radio frequency ablation of spinal neoplasms in late stage cancer disease: A report of three cases. Spine.

[9] Filippiadis DK, Masala S, Lucatelli P, Kelekis A (2022). Update on interventional radiology of the Spine. Semin. Musculoskelet. Radiol..

[10] Godzik J (2019). A quantitative assessment of the accuracy and reliability of robotically guided percutaneous pedicle screw placement: Technique and application accuracy. Oper. Neurosurg. (Hagerstown, Md.).

[11] Yamanouchi K (2023). Validation of a surgical drill with a haptic interface in spine surgery. Sci. Rep..

[12] van Haaren EH (2008). Effect of long-term preservation on the mechanical properties of cortical bone in goats. Acta Orthop..

[13] Mark-10 Force and Torque Measurement. Product Information | Mark-10 Force and Torque Measurement. Available at https://mark-10.com/resources/product-information/ (2020).

[14] Merten N (2019). A two-step risk assessment method for radiofrequency ablations of spine metastases. Comput. Biol. Med..

[15] Roy-Camille, R., Saillant, G. & Mazel, C. Internal fixation of the lumbar spine with pedicle screw plating. *Clin. Orthop. Relat. Res.* 7–17 (1986). 3955999

[16] Kim KJ (2019). Hounsfield units on lumbar computed tomography for predicting regional bone mineral density. Open Med. (Warsaw, Poland).

[17] DICOM. DICOM. Available at https://www.dicomstandard.org/ (2023).

[18] Chan YH (2003). Biostatistics 104: Correlational analysis. Singap. Med. J..

[19] Cohen J (1988). Statistical Power Analysis for the Behavioral Sciences.

[20] Cömert A (2009). Fresh-frozen vs. embalmed bone: Is it possible to use formalin-fixed human bone for biomechanical experiments on implants?. Clin. Oral Implant. Res..

[21] Unger S, Blauth M, Schmoelz W (2010). Effects of three different preservation methods on the mechanical properties of human and bovine cortical bone. Bone.

[22] Berlin C (2020). Erhobene Daten zur Instrumentierung in Freihandtechnik und Literaturvergleich zu fluoroskopisch- und CT-gestützter Navigation. Der Orthopade..

[23] Caprara S (2022). Bone density optimized pedicle screw instrumentation improves screw pull-out force in lumbar vertebrae. Comput. Methods Biomech. Biomed. Eng..

[24] Cowin SC (2001). Bone Mechanics Handbook.

[25] Sedlin ED (1965). A rheologic model for cortical bone. A study of the physical properties of human femoral samples. Acta Orthop. Scand. Suppl..

[26] Weaver JK (1966). The microscopic hardness of bone. J. Bone Jt. Surg. Am. Vol..

[27] Linde F, Sørensen HCF (1993). The effect of different storage methods on the mechanical properties of trabecular bone. J. Biomech..

[28] Panjabi MM, Krag M, Summers D, Videman T (1985). Biomechanical time-tolerance of fresh cadaveric human spine specimens. J. Orthop. Res. Off. Publ. Orthop. Res. Soc..

[29] Lander SL, Brits D, Hosie M (2014). The effects of freezing, boiling and degreasing on the microstructure of bone. Homo: Internationale Zeitschrift fur die vergleichende Forschung am Menschen.

[30] Kaye B (2012). the effects of freezing on the mechanical properties of bone. TOBONEJ.

[31] Alam K (2023). Effect of drill quality on biological damage in bone drilling. Sci. Rep..

[32] Seebeck J (2004). Effect of cortical thickness and cancellous bone density on the holding strength of internal fixator screws. J. Orthop. Res. Off. Publ. Orthop. Res. Soc..

[33] Tang Y-X, Peng S-L, Chen Y-W, Huang H-M, Shih C-T (2023). Evaluating the contact anatomy and contact bone volume of spinal screws using a novel drilled surface image. PloS One.

[34] Lavecchia CE (2018). Lumbar model generator: A tool for the automated generation of a parametric scalable model of the lumbar spine. J. R. Soc. Interface.

[35] Scoles PV, Linton AE, Latimer B, Levy ME, Digiovanni BF (1988). Vertebral body and posterior element morphology: The normal spine in middle life. Spine.

[36] Konermann W, Stubbe F, Link T, Meier N (1999). Axiale Bruchfestigkeit von thorakolumbalen Wirbelkörpern–eine experimentelle biomechanische Studie. Zeitschrift fur Orthopadie und ihre Grenzgebiete.

[37] McBroom RJ, Hayes WC, Edwards WT, Goldberg RP, White AA (1985). Prediction of vertebral body compressive fracture using quantitative computed tomography. J. Bone Jt. Surg. Am..

[38] Kwak M (2022). Mechanical and geometric analysis of fenestration design for polymethylmethacrylate-augmented pedicle screw fixation. Int. J. Spine Surg..

[39] Chan A, Parent E, Narvacan K, San C, Lou E (2017). Intraoperative image guidance compared with free-hand methods in adolescent idiopathic scoliosis posterior spinal surgery: A systematic review on screw-related complications and breach rates. Spine J. Off. J. N. Am. Spine Soc..

[40] Tormenti MJ (2010). Intraoperative computed tomography image-guided navigation for posterior thoracolumbar spinal instrumentation in spinal deformity surgery. Neurosurg. Focus.

[41] Noriega DC (2017). Risk-benefit analysis of navigation techniques for vertebral transpedicular instrumentation: A prospective study. Spine J Off. J. N. Am. Spine Soc..

[42] Li X (2019). Computed tomography measurement of the bone matrix of vertebral pedicle and its clinical significance. Folia Morphol..

