# Supplementary material for: Correlation of CT-based bone mineralization with drilling-force measurements in anatomical specimens is suitable to investigate planning of trans-pedicular spine interventions
Source: Sci Rep. 2024 Jan 18;14:1579. doi: 10.1038/s41598-023-50204-2 (PMC10796759; doi:10.1038/s41598-023-50204-2)

Supplement 2: Scatterplot diagram of Pearson (parametric) rank correlation coefficient sorted by vertebrae (A-F: T12-L5) in individual B. Correlations of normalized force values (N, y-axis) and intensity values (GV, x-axis). For square of Pearson correlation coefficient R^2 (coefficient of determination) also see Table 4.

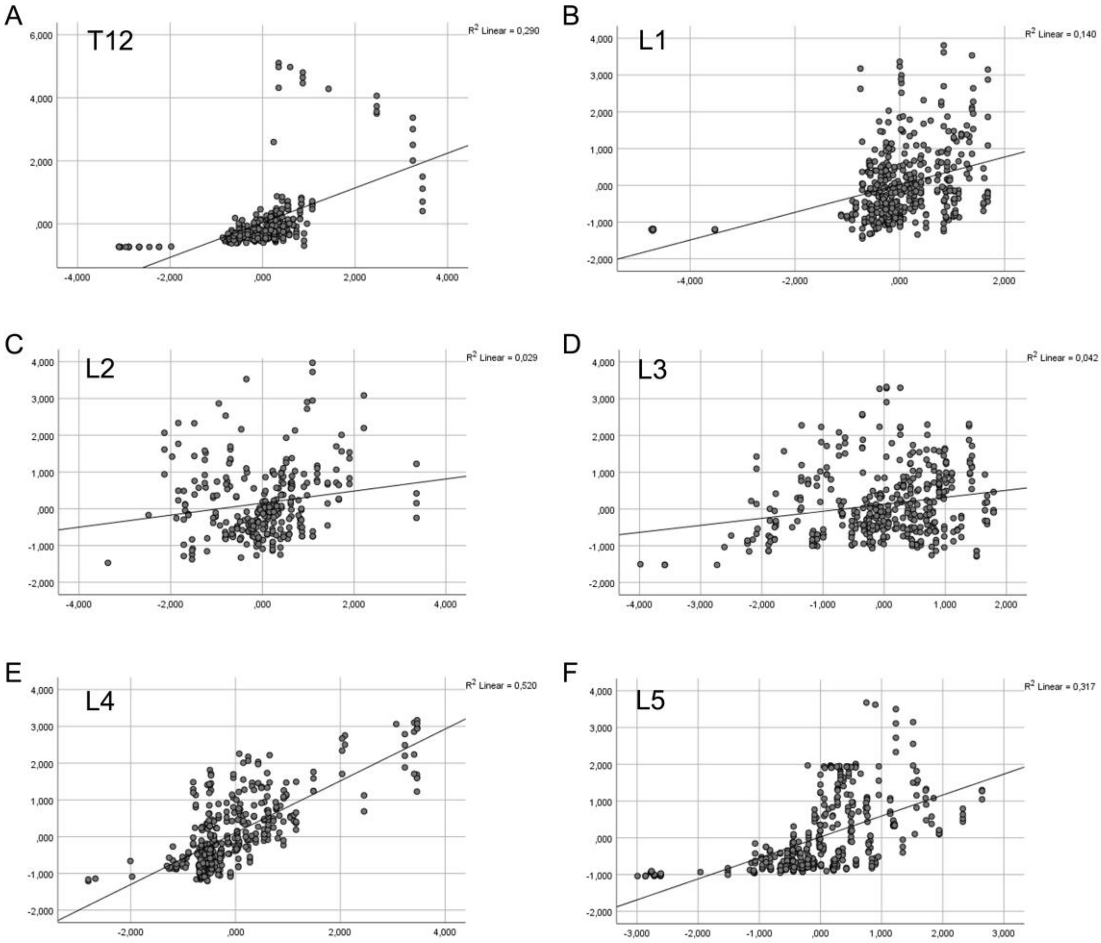

Supplement: Supplementary file 2 — Supplementary Information 2. [file 41598_2023_50204_MOESM2_ESM.pdf]
